# Andrew Kaplan (1959–2006): remembering a friend and a colleague

**DOI:** 10.1186/1742-4690-3-61

**Published:** 2006-09-12

**Authors:** Ronald Swanstrom

**Affiliations:** 1 UNC Center For AIDS Research, University of North Carolina at Chapel Hill, Chapel Hill, North Carolina 27599-7295, USA

## Abstract

A remembrance of Andy Kaplan as a colleague, a friend, and a member of our community.

## Obituary

It is with great sadness that I write of the death of Dr. Andrew Kaplan, Associate Professor of Medicine and of Microbiology and Immunology, University of North Carolina at Chapel Hill, at the age of 47. Andy died suddenly on June 28, 2006 from unrecognized heart disease. We will miss Andy for his warmth, his wit, his intelligence, and his commitment to the retrovirology community.

Andy had strong academic training with an undergraduate degree from Harvard and a medical degree from Columbia. He did his residency training at UNC Chapel Hill then stayed for a fellowship in infectious diseases. I first met him as a fellow when he chose my lab for training in HIV molecular biology.

Those who trained in medicine at this time were among the first group of researchers confronted with the HIV epidemic. Andy's medical school training in New York in the early 1980s brought him into contact with AIDS at a time when little was known and treatment didn't exist. The AIDS Clinical Trials Unit was founded at UNC just as Andy was finishing his residency and becoming an ID Fellow. Both the need and the challenge drew him to this emerging field.

Andy joined my lab as a fellow around 1990. In his initial work he examined the site of processing of the HIV-1 Gag protein. He was able to show that the full range of Gag processing intermediates were present at the plasma membrane, suggesting processing is at least initiated during the budding process. This was a fortuitous time to be working on questions involving the viral protease as the first protease inhibitors were being developed. Due to a generous collaboration with Dr. Dale Kempf at Abbott, Andy was able to carry out some of the first selections for resistance to a viral protease inhibitor, and with the assistance of other members of the lab identified residues within the protease involved in resistance. Further studies with a protease inhibitor included an exploration of the extent of processing inhibition needed to ablate virion infectivity.

In 1993 Andy moved to UCLA as an assistant professor. He continued studies in molecular virology with one example being the novel observation of the discovery of a primary infection case where the transmitted virus carried a deleterious mutation which subsequently reverted. During this time his lab also initiated studies into the nature of the dimer linkage structure of MLV, and his propensity to participate in large collaborative studies started to become apparent with colleagues at UCLA.

Prior to leaving UNC Andy married Carol Golin, an MD researcher interested in issues of adherence to therapy. They found overlapping interests in the study of the relationship of adherence to the development of drug resistance, a line of research for which they received joint funding. These studies also represented a significant expansion of Andy's scientific interests into the area of behavioral science.

We were fortunate to recruit Andy and Carol back to UNC in 1998. Andy continued his studies in molecular virology with research on the HIV protease autoactivation and the dimer interface. He remained deeply steeped in the use of mutagenesis linked to high throughput assays for function to address fundamental questions about the viral protease.

In the last few years Andy's intellectual breadth became fully apparent as did his role as a mentor and collaborator. He made contributions to the development of a larger UNC effort to study acute infections across the entire state. He also provided senior leadership to a novel set of studies following the impact on behavior, therapy and care of incarcerated persons who return to their communities.

Andy was an active member of our research community. He gave freely of his time to serve on any number of study sections. I frequently relied on his commitment to peer review as a reviewer for the Journal of Virology, always grateful for his thoughtful reviews. Over the last year he was one of the most active reviewers for JV. Andy was one of a very small number of MDs who made a point of attending the Cold Spring Harbor Retrovirus Meeting held each May. Presentations at this meeting have been a rite-of-passage for young retrovirologists for thirty years and Andy continued to contribute to this unique and valuable meeting that is an essential gathering of our molecular virology community.

In my mind Andy will always be the person who would go out for a lunchtime run with me at CSH. He was wonderful company with a mind full of curiosity and interest in the topic of the day and happy to carry the conversation on the uphill stretches. After running the obligatory 4 miles down to the beach and back, Andy would deliver me back to Blackford then head out for the rest of his run, ever expecting more of himself.

We have established The Andy Kaplan Memorial Lectureship within the Infectious Diseases Division here at UNC Chapel Hill. For those of you wishing to remember Andy by contributing to this effort please send a check to The Andy Kaplan Fund, mailed to LouAnne Loschin, ID Division CB 7030, Department of Medicine. Rm 2118D Bioinformatics Bldg., University of North Carolina at Chapel Hill, Chapel Hill, NC, 27599.

**Figure 1:**
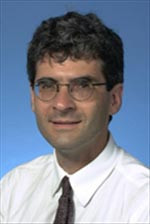
Andy Kaplan, circa 2002.

